# Image-guided interventional radiotherapy (modern brachytherapy) for treatment of vaginal intraepithelial neoplasia: single-institution experience and systematic literature review and meta-analysis

**DOI:** 10.1007/s00066-026-02549-6

**Published:** 2026-05-26

**Authors:** V. Lancellotta, A. P. Solazzo, B. Fionda, G. Garganese, S. M. Fragomeni, A. Federico, M. C. La Milia, P. Dragonetti, E. Rosa, L. Bannoni, M. De Angeli, S. Russi, S. Laurino, A. G. Morganti, R. P. De Vincenzo, G. Macchia, M. A. Gambacorta, R. Iezzi, L. Tagliaferri

**Affiliations:** 1 Radioterapia Oncologica, Dipartimento di Diagnostica per Immagini e Radioterapia Oncologia, Fondazione Policlinico Universitario “A. Gemelli” IRCCS, Rome, Italy; 2 Radiation Oncology Unit, IRCCS-CROB, Rionero in Vulture, Italy; 3 U.O.C. Ginecologia Oncologica, Dipartimento di Scienze della Salute della Donna e del Bambino, Fondazione Policlinico Universitario A. Gemelli IRCCS, Rome, Italy; 4 Gemelli Women’s Health Center for Digital and Personalized Medicine, Dipartimento Scienze della Vita e Sanità Pubblica, Sezione di Ginecologia e Ostetricia, Università Cattolica del Sacro Cuore, Rome, Italy; 5 UOC Fisica per le Scienze della Vita, Dipartimento di Diagnostica per Immagini e Radioterapia Oncologica, Fondazione Policlinico Universitario A. Gemelli IRCCS, Rome, Italy; 6 Department of Theoretical and Applied Sciences, eCampus University, Novedrate, Italy; 7 Laboratory of Preclinical and Translational Research, IRCCS-CROB, Rionero in Vulture, Italy; 8 Radiation Oncology, IRCCS Azienda Ospedaliero-Universitaria di Bologna, Radiation Oncology, Department of Medical and Surgical Sciences (DIMEC), University of Bologna, Bologna, Italy; 9 Unità operativa di Patologia del basso tratto genitale, Divisione di Ginecologia Oncologica, Dipartimento Scienze della Salute della Donna, del Bambino, Fondazione Policlinico Universitario A. Gemelli IRCCS, Rome, Italy; 10 Università Cattolica del Sacro Cuore Sede di Roma, Rome, Italy; 11 Radiation Oncology Unit, Responsible Research Hospital-Campobasso and Catholic University of Sacred Heart-Rome, Rome, Italy; 12 Radiologia Interventistica Avanzata, Dipartimento di Diagnostica per Immagini e Radioterapia Oncologia, Fondazione Policlinico Universitario “A. Gemelli” IRCCS, Rome, Italy

**Keywords:** Vaginal intraepithelial neoplasia, Interventional radiotherapy (modern brachytherapy), Local control, Toxicity

## Abstract

**Purpose:**

Vaginal intraepithelial neoplasia (VaIN) is a rare condition that poses diagnostic and management challenges and carries a significant risk of progression to invasive cancer. Image-guided interventional radiotherapy (IG-IRT, modern brachytherapy) has a high overall success rate, but it is usually reserved for poor surgical candidates and those with multifocal disease or failed prior treatments. This study evaluates the efficacy and safety of IG-IRT in high-grade VaIN by combining a retrospective institutional case series with a systematic review of the available literature.

**Methods:**

We retrospectively analyzed patients with VaIN3 who received IG-IRT with curative intent between January 2019 and May 2025. The OncentraBrachy treatment planning system and a Flexitron (Elekta, Stockholm, Sweden) afterloading machine with a 192-Ir source were used for IG-IRT. The IG-IRT total dose was 40 Gy over eight high-dose-rate (HDR) fractions to achieve 60 Gy equivalent dose in 2‑Gy fractions (EQD2α/β10) to the clinical target volume (CTV). The exact vaginal target was decided based on the site and the number of lesions. Primary study endpoint was local control (LC); secondary endpoints included the rate and severity of acute and late treatment-related toxicity. A systematic review of the literature was conducted according to the PRISMA guidelines to contextualize institutional outcomes within the existing evidence.

**Results:**

A total of 10 patients with high-grade VaIN who were naïve to prior radiotherapy were included in this study. The median follow-up duration was 17 months (7–70 months). The 1‑year actuarial LC and overall survival (OS) rates were 100%. No acute side effects were recorded, while late G2 toxicity (vaginal stenosis 1 and atrophy 4) occurred in four patients. Findings from the systematic review were consistent with the institutional results, supporting high rates of local control and an acceptable toxicity profile with IG-IRT in selected patients.

**Conclusion:**

By integrating institutional experience with a systematic review of the literature, this study supports IG-IRT as an effective and safe definitive treatment option for high-grade VaIN, achieving excellent LC with acceptable toxicity. This combined approach provides a more robust framework for clinical decision-making in a rare disease setting.

**Supplementary Information:**

The online version of this article (10.1007/s00066-026-02549-6) contains supplementary material, which is available to authorized users.

## Introduction

Vaginal intraepithelial neoplasia (VaIN) is a rare premalignant condition of the lower female genital tract, accounting for a small proportion of intraepithelial lesions when compared with cervical and vulvar intraepithelial neoplasia [1]. The reported incidence of VaIN is low, but it has increased over recent decades, likely due to improved screening strategies, prolonged survival of patients treated for cervical cancer, and the widespread use of human papillomavirus (HPV) testing [1, 2]. High-grade VaIN (VaIN3) is of clinical relevance, as it carries a non-negligible risk of progression to invasive vaginal carcinoma, reported in up to 9% of cases in historical series [3].

The management of high-grade VaIN remains challenging and controversial. Surgical excision and laser ablation are commonly employed as first-line treatments; however, these approaches may be associated with high recurrence rates, anatomical distortion, and functional impairment, particularly in cases of multifocal or extensive disease [2, 4]. Topical therapies, including imiquimod and 5‑fluorouracil, have shown variable efficacy and limited tolerability, while repeated local treatments may further compromise vaginal anatomy and quality of life [5]. As a result, there is no universally accepted standard of care, and treatment selection is often individualized based on disease extent, prior treatments, patient comorbidities, and functional considerations [1, 2].

Radiotherapy has traditionally been reserved for selected patients with extensive, multifocal, or recurrent VaIN, or for those who are poor surgical candidates [1, 6]. In this context, image-guided interventional radiotherapy (IG-IRT), also referred to as modern brachytherapy, represents a highly conformal treatment modality that allows precise dose delivery to superficial mucosal targets while sparing adjacent organs at risk. The introduction of three-dimensional imaging, particularly CT and MRI, has substantially improved target volume delineation, applicator positioning, and adapted dose optimization thanks to intensity-modulated techniques, thereby reducing treatment-related toxicity compared with historical two-dimensional techniques [7–9].

Despite these technical advances, the role of IG-IRT in the management of high-grade VaIN remains poorly defined. Available evidence is largely limited to small retrospective series, often heterogeneous in terms of patient selection, dose prescription, and follow-up duration [6, 10]. Moreover, current international guidelines provide only general recommendations regarding the use of radiotherapy in VaIN, reflecting the scarcity of high-quality data in this setting [1].

Given the rarity of vaginal intraepithelial neoplasia (VaIN) and the fragmented nature of the available evidence, a comprehensive synthesis of published data is warranted. To date, evidence supporting the use of interventional radiotherapy in high-grade vaginal intraepithelial neoplasia is limited to small, heterogeneous retrospective series, often based on historical two-dimensional techniques and lacking systematic synthesis. To address this unmet need, the present study combines two complementary approaches: an analysis of a single-institution retrospective series of patients with high-grade VaIN treated with image-guided interventional radiotherapy (IG-IRT) and a systematic review of the literature conducted in accordance with the Preferred Reporting Items for Systematic Reviews and Meta-Analyses (PRISMA) guidelines. In the absence of prospective randomized trials, this combined institutional analysis and systematic review represents the highest level of evidence currently achievable to inform clinical decision-making in this rare disease setting [11].

The primary objective is to evaluate the oncological outcomes and treatment-related toxicity of IG-IRT in this rare clinical setting, integrating real-world institutional experience with the existing evidence base.

## Materials and methods

We retrospectively analyzed patients with VaIN3 who received image-guided interventional radiotherapy (IG-IRT) with curative intent between January 2019 and May 2025. The OncentraBrachy treatment planning system and a Flexitron (Elekta, Stockholm, Sweden) afterloading machine with a 192-Ir source were used for IG-IRT treatment. The IG-IRT total dose was 40 Gy over eight high-dose-rate (HDR) fractions to achieve 60 Gy equivalent dose in 2‑Gy fractions (EQD2α/β10) to the clinical target volume (CTV) to 5 mm below the surface of the vaginal mucosa. Before IG-IRT, work-up included biopsy, colposcopy, clinical examination, and thoracic–abdominal–pelvic CT. All patients underwent colposcopy in order to define the exact target of the vagina to be included in the treatment field. The final target was discussed case by case with a gynecologist. The bladder, rectum, and small bowel were contoured as organs at risk and accounted for during treatment planning. Following applicator reconstruction, dwell positions and dwell times were manually optimized to achieve the prescribed dose while respecting organ-at-risk constraints.

Follow-up evaluations were scheduled 6 weeks after treatment completion, every 3 months during the first 2 years, and every 6 months thereafter. Vaginal cytology was performed twice per year, while vaginoscopy and biopsy were undertaken when recurrence was suspected. Acute and late toxicity were assessed using the Common Terminology Criteria for Adverse Events (CTCAE), version 5.0.

The primary study endpoint was local control (LC), while secondary endpoints included the rate and severity of acute and late toxicities. Actuarial outcomes were analyzed using the Kaplan–Meier method. Statistical analysis was carried out using SPSS statistical software (IBM Corp. Version 20.0. Armonk, NY).

This study was conducted within the framework of the Consortium for Brachytherapy Data Analysis initiative, in accordance with its standardized methodological guidelines. Data collection included patient demographics, histological characteristics, radiotherapy technical and dosimetric parameters, treatment-related acute and late toxicities, follow-up duration, and clinical outcomes (Table 1).

**Table 1:** Study characteristics

Author	Year	Study	N0 patients	Median age	IRT technique	Total dose	Acute toxicity	Late toxicity	Local control	FUP	Main results
Woodman et al. [12]	–	Retro	11	39 (34–65)	2D-LDR	36 Gy (24.48–40.80)	Vaginal discharge G2 (3 patients) Diarrhea G2 (3 patients) Cystitis G2 (1 patient)	Dyspareunia (2 patients)	2‑year: 100%	26 (16–36)	IRT as exclusive treatment after surgery Target: 1 patient whole length of the vagina
MacLeod et al. [13]	1985–95	Retro	14	62 (42–85)	2D-HDR	42.5 Gy (34–45 Gy)	Vaginitis G2 (11 patients) Dysuria G2 (11 patients)	Atrophy G2 (12 patients) Fibrosis G2 (12 patients) Telangiectasia G2 (12 patients) Vaginal atrophy G3 (2 patients) Stenosis G3 (2 patients)	5‑year: 86%	43 (6–115)	7 had previously received ablative therapy, 5 with laser, 1 with diathermy, and 1 with 5‑FU cream Target: all patients whole length of the vagina
Ogino et al. [14]	–	Retro	7	52 (44–61)	2D-HDR	23.3 Gy (15–30 Gy)	Rectal bleeding (1 patient)	Stenosis G2 (1 patient)	5‑year: 100%	62 (51–125)	All patients underwent surgery and no patients had previously received ablative therapy
Teruya et al. [15]	1983–98	Retro	13	62 (47–75)	2D-HDR	36 Gy (30–40 Gy)	Vulvovaginitis G1 (1 patient) Urethrocystitis G1 (1 patient)	Atrophy G3 (2 patients) Fibrosis G3 (2 patients) Telangiectasia G3 (2 patients)	10-year: 100%	126.5 (35–218)	No patient had any previous treatment for the present disease
Graham et al. [16]	1985–04	Retro	22	56 (37–70)	2D-PDR	48 Gy (22–52 Gy)	Proctitis G2 (1 patient)	Stenosis G3 (4 patients) Urethral stricture G3 (1 patient) Ulceration G4 (1 patient)	5‑year: 82% 10-year: 82%	77 (32–220)	3 patients had previous laser therapy and 1 patient underwent repeated laser therapy followed by partial vaginectomy
Blanchard et al. [17]	1985–08	Retro	28	63 (38–80)	2D-LDR	60 Gy	Cystitis G1(1 patient) Vaginal mucosal changes G1 (18 patients)	Proctitis G2 (1 patient)	5‑year: 93% 10-year: 93%	41 (0–280)	11 patients had previous conization, 2 patients partial colpectomy, 2 patients laser ablation, 1 patient cryotherapy and interferon therapy
Zolciak-Swińska et al. [18]	2003–13	Retro	20	57 (28–80)	12 patients 2D-HDR 8 patients 3D-HDR	22.5–37.5 Gy	–	Vaginal discharge G2 (2 patients) Dyspareunia G2 (7 patients) Mucositis G3 (6 patients) Stenosis G3 (7 patients) Vaginitis G3 (4 patients)	3‑year: 90%	39 (14–115)	Target: 1/3 upper part of vagina with cervix if exists (6 patients), whole length of vagina (14 patients) 4 patients had radical EBRT In groups of patients irradiated with EQD2 of 47.3–63 Gy and 70 Gy, the risk of severe or moderate toxicity amounted to 16.7% and 71.4%, respectively

*5‑FU* 5-fluorouracil; *EBRT* external-beam radiotherapy; *EQD2* equivalent dose in 2‑Gy fractions; *G* grade; *HDR* high-dose-rate; *IRT* interventional radiotherapy; *LDR* low-dose-rate; *retro* retrospective

### Study design and reporting standards

The systematic review was designed and conducted according to the PRISMA 2020 guidelines. The methodological framework was defined a priori, with explicit specification of the research question using the SPIDER framework, with sample (S), phenomena of interest (PI), design (D), evaluation (E), and research type (R) corresponding to vaginal intraepithelial neoplasia (S), interventional radiotherapy (PI), named types of qualitative data collection and analysis (D), local control and toxicity (E), and qualitative method (R).

### Eligibility criteria

Studies were considered eligible if they met the following criteria: (i) patients with histologically confirmed VaIN; (ii) full text available in English; (iii) timeframe between 1980 and 2025; (iv) reporting of at least one predefined outcome of interest. Case reports with fewer than three patients, review articles, editorials, conference abstracts without full-text availability, and studies not reporting clinical outcomes were excluded.

### Information sources and search strategy

**Fig. 1:**
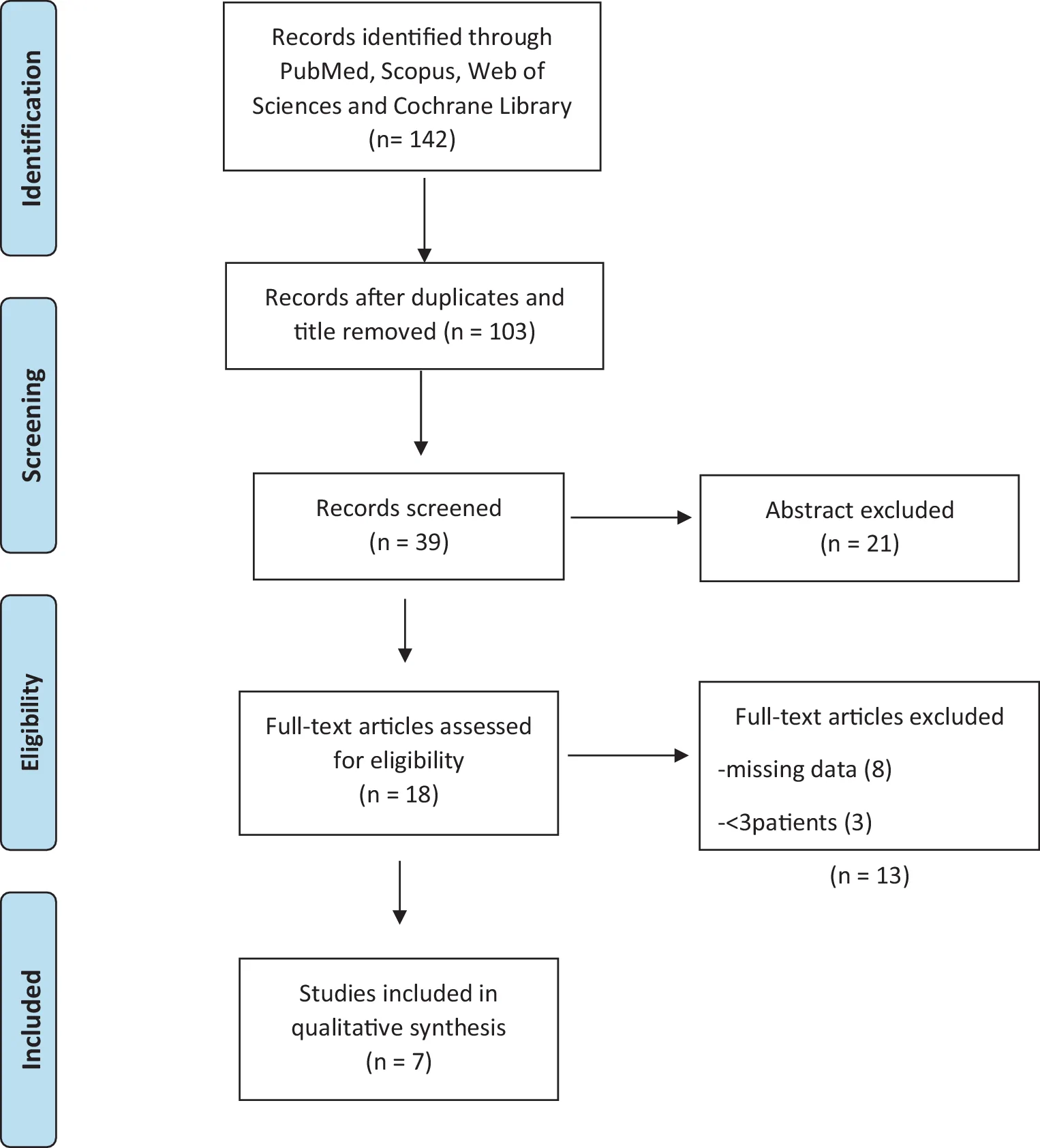
Preferred Reporting Items for Systematic Reviews and Meta-Analyses (PRISMA) flowchart for outcomes and toxicity

A systematic literature search was performed in PubMed/MEDLINE, Scopus, and Web of Science databases. The search strategy combined controlled vocabulary and free-text terms related to VaIN and interventional radiotherapy. Studies were identified through a systematic search of the MEDLINE (PubMed) database using a combination of Medical Subject Headings (MeSH) terms and free-text keywords related to vaginal intraepithelial neoplasia and interventional radiotherapy. The final MEDLINE search string (Fig. 1) was: (“Vaginal Intraepithelial Neoplasia”[All Fields] OR “VaIN”[All Fields]) AND (“Radiotherapy”[MeSH] OR “radiation therapy”[All Fields]) AND (“Brachytherapy”[MeSH] OR “interventional radiotherapy”[All Fields]) AND (“Toxicity”[MeSH] OR “toxicity”[All Fields]).

### Study selection

Titles and abstracts were independently screened by two reviewers (VL, APS) with expertise in gynecologic oncology and radiotherapy. Full-text articles were retrieved and assessed for eligibility. Any discrepancies were resolved through consensus discussion, with involvement of a third reviewer when necessary.

### Data collection and data item

Data extraction was performed independently by two investigators using a predefined standardized data collection form. Extracted variables included: (i) study characteristics (year of publication, study design, sample size); (ii) patient demographics and disease characteristics; (iii) interventional radiotherapy technique (HDR or PDR); (iv) vaginal treatment length; (v) prescribed dose and fractionation schedule; (vi) follow-up duration; (vii) local control; (h) acute and late toxicity graded according to CTCAE or RTOG criteria.

### Risk of bias assessment

The methodological quality and risk of bias of the included studies were assessed after study selection and data extraction. The choice of the appropriate quality-assessment tool was determined based on the design of the included studies. For randomized controlled trials, the Cochrane risk-of-bias tool was planned to be used, while for non-randomized studies, a validated tool for observational studies was applied. The quality assessment evaluated domains including patient selection, comparability of study groups, clarity of intervention description, outcome assessment, adequacy of follow-up, and completeness of reporting. The results of the quality assessment were considered in the interpretation of both qualitative and quantitative analyses.

### Effect measures

Effect measures included proportions for local control, recurrence, progression to invasive disease, and toxicity rates. When applicable, effect sizes were expressed as pooled proportions or risk estimates with corresponding 95% confidence intervals.

### Data synthesis and statistical analysis

Data synthesis included both qualitative and quantitative approaches. A descriptive qualitative synthesis was performed to summarize study characteristics, patient populations, treatment techniques, dose and fractionation schedules, and reported oncological and toxicity outcomes. Meta-analyses were conducted for single-arm studies using pooled proportions, as no comparative arms were available. The evaluation was performed using R statistical software (R Foundation for Statistical Computing, Vienna, Austria) utilizing the *meta* package. As the studies included were single-arm cohorts, pooled estimates for proportions (objective response and toxicity rates) were calculated. To stabilize the variance and ensure the data conformed to normal distribution assumptions, the raw proportions were transformed using the arcsine transformation. A random-effects model was employed for data synthesis to account for between-study variation. The between-study variance (tau^2^) was estimated using the restricted maximum likelihood (REML) method, and the confidence intervals (CIs) for the pooled estimates were adjusted using the Hartung–Knapp (HK) method, which provides more robust estimates when the number of studies is small. Statistical heterogeneity was quantified using the I^2^ statistic and assessed using Cochran’s Q test, with I^2^ values > 50% indicating substantial heterogeneity. To investigate potential sources of heterogeneity, subgroup analyses were performed based on the treatment technique (HDR vs. LDR). Studies reporting zero events were handled by adding a continuity correction only to the zero-cell studies. Results were visualized using forest plots, displaying individual study estimates along with the pooled random-effects estimate and 95% confidence intervals.

### Assessment of reporting bias

Potential reporting bias was evaluated through qualitative assessment of outcome reporting consistency across studies. When enough studies were available, funnel plot asymmetry was assessed to explore potential publication bias.

### Certainty of evidence

The certainty of evidence for each outcome was assessed using the Grading of Recommendations Assessment, Development, and Evaluation (GRADE) approach, considering study limitations, inconsistency, indirectness, imprecision, and publication bias.

## Results

From our database, 10 patients affected by high-grade vaginal intraepithelial cancer were considered in this analysis. All patients had a previous hysterectomy for cervical cancer and did not undergo radiotherapy. The median time from surgery to relapse was 42 months (range 7–60). Previous treatment for VaIN3 included conization in 2 patients and topical treatment with imiquimod in one patient. The median follow-up duration was 17 months (7–70 months), and the median age was 65.5 years (range 44–80 years). All patients received IG-IRT with a total dose of 40 Gy over eight fractions using a multichannel applicator.

The 1‑year actuarial LC and OS rates were both 100%. No acute > G2 toxicity was recorded in any patient, while late G2 toxicity occurred in four patients (vaginal stenosis 1 and atrophy 4).

The median CTV was 23.16 cm^3^ (range 16.52–57.06 cm^3^). The median D90 CTV and D95 CTV were 102.54% (range 99.78%–115.87%) and 99.14% (96.13%–100.86%). The median minimum dose delivered to the most irradiated 2 cm^3^ of rectum, bladder, and small bowel was 45.3 Gy (range 21.8–52.4 Gy), 46.9 Gy (range 13.1–59.4 Gy), and 33.25 Gy (range 5.1–53 Gy), respectively.

### Study selection

The database search identified a total of 142 records. After removal of 39 duplicates, 103 records were screened based on titles and abstracts. Of these, 85 records were excluded because they were not relevant to the research question or did not address high-grade vaginal intraepithelial neoplasia. The full texts of 18 articles were assessed for eligibility. Following full-text review, 11 studies were excluded for the following reasons: absence of clinical outcome data (*n* = 8) and inclusion of fewer than three patients (*n* = 3). At the end of the process, seven retrospective studies fulfilled the inclusion criteria [12–18], including 129 patients treated with interventional radiotherapy.

### Study characteristics and results of individual studies

Across the seven included studies, interventional radiotherapy was delivered using different techniques: low-dose-rate (LDR) [12, 17] or pulsed-dose (PDR) IRT [16] were used in three studies, whereas high-dose-rate (HDR) IRT was used in four [13–15, 18]. Only one study performed three-dimensional CT-guided IRT in 8 patients [18]. Prescribed doses and fractionation schedules were highly heterogeneous among studies, with total doses generally between approximately 36 and 60 Gy and fraction sizes varying from 3 to 6 Gy [12–18]. Target volume definitions also varied, with some studies treating the entire vaginal length [12, 13, 18] and others adopting a more focal approach [18].

Median follow-up varied widely across studies, ranging from approximately 26 months to over 10 years (median follow-up 43 months), allowing assessment of both oncologic outcomes and late toxicity in all reports [12, 18]. Median local control at 5 years was 89.5% (range 82%–100%) [13, 14, 16, 17]. Progression to invasive vaginal cancer was rare across all series [14, 16, 18].

More frequent G1–G2 acute toxicities involved vaginal mucosa (19 patients G1 and 14 patients G2) [12, 13, 15, 17]. One study reported rectal bleeding in one patient [14]. Vaginal late G3 toxicities were reported in 31 patients (atrophy 4 patients [13, 15], stenosis 13 patients [13, 16, 18], fibrosis 2 patients [15], telangiectasia 2 patients [15], vaginitis 10 patients [18]), and G4 in one patient (vaginal ulceration [16]).

### Risk of bias in studies and reporting bias

After reviewing the quality of each study, all seven studies [12–18] were found to have a very high risk of bias (Supplementary Table 1 and Supplementary Table 2).

### Results of synthesis

**Fig. 2:**
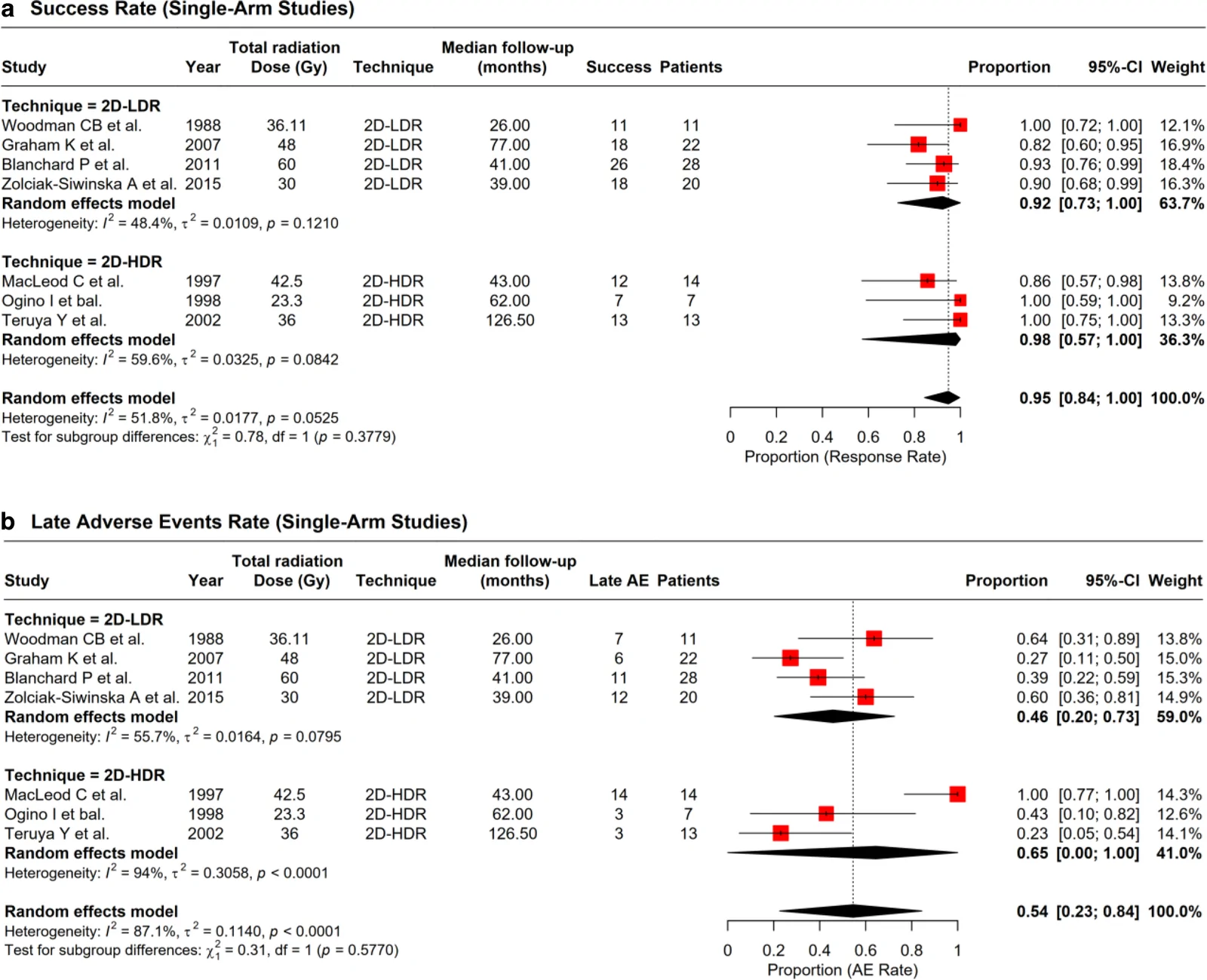
Analysis of success rate (local control) and toxicity. *2D* two-dimensional, *Gy* gray, *HDR* high dose rate, *CI* confidential index, *LDR* low dose rate

Seven single-arm studies comprising 129 patients were included in the meta-analysis to evaluate the success rate (SR; Fig. 2a). Due to significant heterogeneity (Q = 12.46, df = 6, *p* = 0.0525; I^2^ = 51.8%), a random-effects model (Hartung–Knapp adjustment) was employed. The pooled SR was 94.8% (95% CI: 84.43–99.70%). Overall, these findings confirm that interventional radiotherapy achieves high rates of local control across different historical techniques, although substantial heterogeneity reflects differences in treatment era, planning approach, and dose prescription. Subgroup analysis was performed to assess potential differences based on the technique used (2D high-dose-rate [2D-HDR] vs. 2D low-dose-rate [2D-LDR]). The 2D-HDR subgroup (k = 3) demonstrated a success rate of 97.97% (95% CI: 57.33%–100.00%), while the 2D-LDR subgroup (k = 4) exhibited a rate of 92.30% (95% CI: 72.77%–99.98%). The test for subgroup differences revealed no statistically significant distinction between the two techniques (*p* = 0.38).

Regarding the occurrence of late adverse events (AEs; Fig. 2b), the pooled estimate using a random-effects model was 54.49% (95% CI: 22.60%–84.44%). There was substantial heterogeneity observed across the included studies (I^2^ = 87.1%, 95% CI: 75.7%–93.2%; Q = 46.59; *p* < 0.0001). Following subgroup analysis based on the technique used (2D-HDR vs. 2D-LDR), the pooled event rate in the 2D-HDR group was 64.54% (95% CI: 0.00%–100.00%) with high heterogeneity (I^2^ = 94.0%), whereas for the LDR technique, the pooled event rate was 45.77% (95% CI: 20.28%–72.52%) with moderate heterogeneity (I^2^ = 55.7%). While the 2D-HDR group demonstrated a higher proportion of adverse events compared to the 2D-LDR group, the test for subgroup differences using the random effects model was not statistically significant (*p* = 0.58), suggesting that the technique used may not fully explain the observed heterogeneity.

### Certainty of evidence

The certainty of evidence was assessed using the GRADE approach and was judged to be low to very low for the evaluated outcomes. For success rate (local control), the certainty was rated as low, as all included studies were retrospective, single-arm series. The evidence was downgraded for risk of bias, indirectness related to heterogeneous techniques and historical cohorts, and imprecision due to small sample sizes, despite generally consistent local control rates across studies. For toxicity, the certainty of evidence was considered very low. In addition to the limitations, toxicity outcomes were further downgraded for serious inconsistency stemming from heterogeneous and incomplete reporting, variable grading systems, and predominantly descriptive outcome measures. Small cohorts and potential underreporting of late adverse events contributed to substantial imprecision. Overall, while the available data suggest favorable local control with acceptable toxicity, the low certainty of evidence warrants cautious interpretation.

## Discussion

This study provides a comprehensive evaluation of interventional radiotherapy for treatment of high-grade vaginal intraepithelial neoplasia, combining a contemporary institutional experience with a systematic review and meta-analysis of the available literature. Overall, our findings confirm that interventional radiotherapy represents a highly effective local treatment modality for VaIN 3, while also highlighting the critical issue of treatment-related vaginal toxicity and the potential role of modern image guidance and supportive care in improving the therapeutic ratio.

In the institutional cohort, exclusive IG-IRT achieved excellent oncological outcomes, with complete local control observed in all patients and no disease-related deaths during follow-up. These results are particularly noteworthy given the challenging clinical context of VaIN 3, which often presents as multifocal disease and frequently occurs in previously treated or surgically altered vaginal anatomy. Importantly, no acute toxicity was observed, and late adverse events were limited to low-grade vaginal morbidity. Although follow-up remains relatively short, these findings suggest that modern IG-IRT can provide effective disease eradication with acceptable toxicity when delivered in experienced centers. When contextualized within the existing literature, our institutional outcomes compare favorably with previously published series [12–18]. The systematic review and meta-analysis demonstrate that interventional radiotherapy achieves consistently high rates of local control across studies, with pooled estimates exceeding 95%, irrespective of the dose rate employed. These results are in line with earlier reports showing that radiotherapy is highly effective in controlling intraepithelial vaginal disease, particularly when surgical excision is limited by lesion extent, anatomical constraints, or prior treatments [6, 12–18]. However, the meta-analysis also revealed a non-negligible incidence of late grade ≥ 3 toxicity, with substantial heterogeneity across studies and higher toxicity rates reported in some HDR series. Importantly, most severe late toxicities reported in the literature derive from historical two-dimensional brachytherapy series, whereas contemporary image-guided interventional radiotherapy appears to be associated with a more favorable toxicity profile. In this context, the markedly favorable toxicity profile observed in our cohort deserves attention. Compared with the pooled literature data, our patients experienced lower rates of severe late vaginal toxicity while maintaining excellent local control. This difference may plausibly be attributed to the systematic use of image-guided interventional radiotherapy, with CT-based planning and individualized target volume definition in all patients. Image guidance likely enabled improved dose conformity and more accurate sparing of surrounding normal tissues, thereby minimizing unnecessary irradiation of healthy vaginal mucosa. In addition, all patients received structured counselling and proactive supportive care measures aimed at toxicity reduction, including the regular use of vaginal moisturizers or ovules, vaginal irrigations, and vaginal dilators [20, 21]. Such preventive strategies are inconsistently reported in historical series and may have contributed to the reduced incidence of late adverse events observed in our cohort.

Several limitations must also be acknowledged. First, the institutional cohort is limited by its small sample size and relatively short follow-up, which restricts the ability to draw firm conclusions regarding long-term disease control and late toxicity. Nevertheless, available evidence suggests that most recurrences of high-grade VaIN occur within the first 12–24 months, partially mitigating the impact of limited follow-up on early oncological outcome assessment. Although VaIN is a rare condition, these factors inherently limit statistical power and preclude subgroup analyses. Second, all studies included in the systematic review were retrospective, single-arm series, with substantial heterogeneity in terms of treatment techniques, dose prescription, target volume definition, and toxicity reporting. This heterogeneity is reflected in the high I^2^ values observed in the pooled analyses and reduces the precision of summary estimates. Moreover, the lack of comparative data prevents any definitive conclusions regarding the superiority of one interventional radiotherapy technique over another. Third, toxicity reporting across studies was inconsistent and often incomplete, with different grading systems and variable follow-up durations. As a result, the true incidence of late vaginal morbidity may be underreported in the literature, and comparisons across series should be interpreted with caution. Finally, although the favorable toxicity profile observed in our cohort is encouraging, it may reflect the experience of a specialized center with expertise in image-guided interventional radiotherapy and comprehensive supportive care. Therefore, the generalizability of these findings to lower-volume or less specialized settings may be limited.

## Conclusion

Given the rarity of high-grade vaginal intraepithelial neoplasia and the absence of standardized treatment pathways, management should be discussed within a dedicated multidisciplinary tumor board to ensure appropriate patient selection and personalized treatment planning. Image-guided interventional radiotherapy provides excellent local control with acceptable toxicity in high-grade vaginal intraepithelial neoplasia. Given the rarity of the disease and the heterogeneity of treatment options, management should be discussed within a multidisciplinary tumor board to ensure a personalized therapeutic approach. Prospective randomized studies are needed to better define optimal treatment strategies.

## Supplementary Information

ESM1: Supplementary material 1

ESM2: Supplementary material 2

ESM3: Supplementary material 3

## Data Availability

All data supporting the findings of this work are included in the article.
