# Supplementary material for: Image-guided interventional radiotherapy (modern brachytherapy) for treatment of vaginal intraepithelial neoplasia: single-institution experience and systematic literature review and meta-analysis
Source: Strahlenther Onkol. 2026 May 26;202(9):976–85. doi: 10.1007/s00066-026-02549-6 (PMC13518449; doi:10.1007/s00066-026-02549-6)
Supplement: Supplementary file 1 [file 66_2026_2549_MOESM1_ESM.docx]

| Study design | Risk of bias | Inconsistency | Indirectness | Imprecision | N. of patients | | Effect | | | Certainty |
| --- | --- | --- | --- | --- | --- | --- | --- | --- | --- | --- |
|  |  |  |  |  |  | | Relative  (95% CI) | Absolute  (95% CI) | |  |
| **LC (Woodman; British Journal of Obstetrics and Gynaecology 1988) (follow-up: median 26 months)** | | | | | | | | | | |
| Observational (retrospective) | Serious^a^ | Not serious | Serious^b^ | Serious^c^ | 11 | Not estimable | | | 85% | Low |
| **LC (MacLeod; Gynecolocic Oncology 1997) (follow-up: median 43 months)** | | | | | | | | | | |
| Observational (retrospective) | Serious^a^ | Not serious | Serious^d^ | Serious^c^ | 14 | Not estimable | | | 90% | Low |
| **LC (Ogino; International Journal of Radiation Oncology Biology Physics 1998) (follow-up median 62 months)** | | | | | | | | | | |
| Observational (retrospective) | Serious^a^ | Not serious | Serious^b^ | Serious^c^ | 7 | Not estimable | | | 100% | Low |
| **LC (Teruya; American Journal of Obstetrics & Gynecology: Home Page 2002) (follow-up: median 126.5 months)** | | | | | | | | | | |
| Observational (retrospective) | Serious^a^ | Not serious | Serious^b^ | Serious^c^ | 13 | Not estimable | | | 92% | Low |
| **LC (Graham; Gynecolocic Oncology 2007) (follow-up: median 77 months)** | | | | | | | | | | |
| Observational (retrospective) | Serious^a^ | Not serious | Serious^d^ | Serious^c^ | 22 | Not estimable | | | 82-86% | Low |
| **LC (Blanchard; The Oncologist 2011) (follow-up median 41 months)** | | | | | | | | | | |
| Observational (retrospective) | Serious^a^ | Not serious | Serious^b^ | Serious^c^ | 28 | Not estimable | | | 95% | Low |
| **LC (Zolciak-Swińska; European Journal of Obstetrics & Gynecology and Reproductive Biology 2015) (follow-up median 39 months)** | | | | | | | | | | |
| Observational (retrospective) | Serious^a^ | Not serious | Serious^d^ | Serious^c^ | 20 | Not estimable | | | 90-95% | Low |

*Abbreviation: LC: local control; N: number.*

**Explanations:**

**^a^**retrospective, single-arm, selection bias

^b^obsolete Radiotherapy techniques

^c^small sample size

^d^heterogeneous dose/targets
